# Evidence for similar early but not late representation of possible and impossible objects

**DOI:** 10.3389/fpsyg.2015.00094

**Published:** 2015-02-16

**Authors:** Erez Freud, Bat-Sheva Hadad, Galia Avidan, Tzvi Ganel

**Affiliations:** ^1^Department of Psychology, Ben-Gurion University of the Negev, Beer-Sheva, Israel; ^2^Zlotowski Center for Neuroscience, Ben-Gurion University of the Negev, Beer-Sheva, Israel; ^3^Edmond J. Safra Brain Research Center, Faculty of Education, University of Haifa, Haifa, Israel

**Keywords:** impossible objects, coarse-to-fine, object-based attention, 3D structure, object recognition

## Abstract

The perceptual processes that mediate the ability to efficiently represent object 3D structure are still not fully understood. The current study was aimed to shed light on these processes by utilizing spatially possible and impossible objects that could not be created in real 3D space. Despite being perceived as exceptionally unusual, impossible objects still possess fundamental Gestalt attributes and valid local depth cues that may support their initial successful representation. Based on this notion and on recent findings from our lab, we hypothesized that the initial representation of impossible objects would involve common mechanisms to those mediating typical object perception while the perceived differences between possible and impossible objects would emerge later along the processing hierarchy. In Experiment 1, participants preformed same/different classifications of two markers superimposed on a display containing two objects (possible or impossible). Faster reaction times were observed for displays in which the markers were superimposed on the same object (“object-based benefit”). Importantly, this benefit was similar for possible and impossible objects, suggesting that the representations of the two object categories rely on similar perceptual organization processes. Yet, responses for impossible objects were slower compared to possible objects. Experiment 2 was designed to examine the origin of this effect. Participants classified the location of two markers while exposure duration was manipulated. A similar pattern of performance was found for possible and impossible objects for the short exposure duration, with differences in accuracy between these two types of objects emerging only for longer exposure durations. Overall, these findings provide evidence that the representation of object structure relies on a multi-level process and that object impossibility selectively impairs the rendering of fine-detailed description of object structure.

## INTRODUCTION

Throughout our daily lives we recognize thousands of objects quickly and accurately. One particular requirement for successful object recognition is the extraction of object 3D structure, which enables humans to recognize objects despite enormous changes across viewpoint and size (Marr, 1982). To date, the exact nature of the cognitive and neural mechanisms that support the representation of object 3D structure is still not fully understood.

**FIGURE 1:**
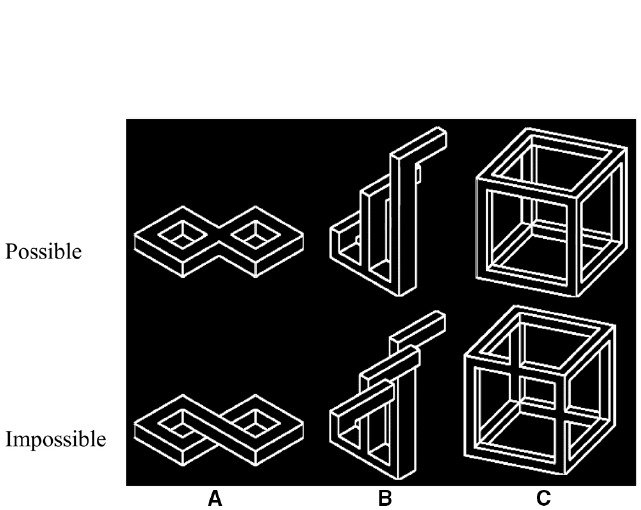
**Possible (upper row) and impossible objects (bottom row) that were used in Experiment 1.** Note, that the two sets of objects were matched so that only relatively small physical differences would establish a perception of spatial impossibility. Stimuli contained one **(A)** or two **(B,C)** structural violations that induced object impossibility.

In the present study we utilized a special type of visual illusion known as “impossible objects” to further unravel the perceptual processes that mediate the representation of object 3D structure. Impossible objects are defined as 2D drawings that represent 3D objects that could not exist in real 3D space (Penrose and Penrose, 1958; Figure 1). At the 2D level, the physical differences between possible and impossible objects are minor; however, the 3D interpretation of the structure of these two object categories is markedly different. Hence impossible objects offer a unique opportunity to test the representation of object 3D structure while other visual properties are well controlled.

Impossible objects reflect internal inconsistencies between the global and local information embedded in the stimulus. Specifically, while the local elements that compose the object are completely valid, the perceived global 3D structure is incoherent. Such inconsistencies were previously investigated in the context of different depth cues such as structure from motion, disparity and perspective lines (e.g., Domini et al., 1998, 2006; Di Luca et al., 2004; Knill and Pouget, 2004; Burge et al., 2005; Knill, 2007). For example, Di Luca et al. (2004) found that the perception of local surfaces that were defined by 3D structure from motion, were biased by the global properties of the stimuli. These findings strongly suggest that the perceived 3D shape is generated based on the integration of various cues, and that the effect of global information can be considered as automatic in nature. Accordingly, previous studies that investigated the perceptual processes that mediate the representation of object impossibility have found that the visual system is highly susceptible to detect object impossibility even when it is task irrelevant. In particular, it was found that a set of cortical regions along the dorsal and ventral visual streams is highly sensitive to object impossibility (Freud et al., 2015). Additionally, a behavioral study showed that 4 month old infants looked longer at impossible objects compared to possible objects (Shuwairi et al., 2007). Similar sensitivity was found even in non-human animals (i.e., newly hatched chicks) that exhibited a spontaneous preference to possible objects (Regolin et al., 2011).

On the other hand, other studies also showed similarities in the perceptual and neural mechanisms that mediate the representations of possible and impossible objects. Specifically, similar Garner interference effects (i.e., worse performance in a classification task of a given object’s dimension when an irrelevant dimension is varied; Garner and Felfoldy, 1970) was found for possible and impossible objects, suggesting that the two object categories were processed in a holistic fashion (Freud et al., 2013a). Additionally, in fMRI studies, similar adaptation patterns were found for possible and impossible objects (Habeck et al., 2006; Freud et al., 2013b).

These seemingly contradicting findings imply that under some conditions, the visual system is insensitive to object impossibility, however, under other conditions, it may still retain great susceptibility to object 3D structure and to the inconsistencies between the global and local information. Importantly, as noted above, impossible objects, just as possible ones, possess valid depth cues which are based on fundamental Gestalt shape attributes (e.g., object closure, distinguishable surfaces, and volume properties) (Koffka, 1935; Kovacs and Julesz, 1993; Geisler et al., 2001). Thus, it is reasonable to assume that initial representations of these objects could be successfully generated. Consequently, the spatial ambiguity embedded in impossible objects may impair higher perceptual processes.

We recently tested this view in the context of a behavioral studies focused on the role of spatial frequency in the representation of possible and impossible objects. These studies already alluded to a dissociable role of different spatial frequency ranges in object recognition. Specifically, it has been demonstrated that low spatial frequency (LSF) information conveys global properties of the objects and supports the generation of their initial coarse representation, while high spatial frequency (HSF) information is processed later in time and supports the rendering of fine-grained description of these objects (e.g., Sugase et al., 1999; Bar, 2003; Bar et al., 2006). Consistently, using the Garner speeded classification task (Garner and Felfoldy, 1970) we showed that a similar processing style (i.e., holistic representation) was applied to both possible and impossible objects following the filtering of their HSF content. Contrary, when LSF information, which mainly supports the initial representation, was filtered out, the responses to the two object categories were dissociated. Particularly, possible objects were still processed holistically, while impossible objects were processed in an analytic—local fashion and were no longer perceived as unified objects (Freud et al., 2014).

The current study was aimed to further investigate the perceptual processes that mediate the representation of object 3D structure. In two experiments, we tested two basic predictions stemming from our working hypothesis. Experiment 1 tested whether impossible objects are organized and perceived as unified wholes similarly to possible objects using an established perceptual measure of object-based processing style. Experiment 2 further examined whether the observed differences between object categories rely on early or on late perceptual processes.

## EXPERIMENT 1

The purpose of Experiment 1 was to test whether possible and impossible objects share similar initial perceptual processes using an established behavioral paradigm. Specifically, we applied the classical “object-based attention” paradigm originally devised by Duncan (1984). In this paradigm participants are asked to perform simple perceptual classifications of two visual elements in the display (e.g., same/different classifications) and the typical finding is of better performance when the two to be judged features, belong to the same object rather than placed on two different objects. The common interpretation of this result is that visual attention operates on object-based representations, and is therefore constrained by perceptual organization processes of object segregation (Duncan, 1984; Kimchi et al., 2007).

The main hypothesis was that impossible objects would have a similar object-based processing style as possible ones. Specifically, we hypothesized that if the visual system constructs a valid initial representation for impossible objects, similarly to possible objects, these perceptually organized representations should capture attention so that targets placed on the same object would be more efficiently processed than when placed on two different impossible objects.

### MATERIALS AND METHODS

#### Participants

Thirty students with normal or corrected to normal vision participated in the experiment. They all provided informed consent to participate in the experiment and received $5 equivalent for their participation. The data of one participant was discarded from the analysis due to exceptionally low accuracy (less than 70% compared to average of 93.6% of the other participants). All experimental procedures were approved by the ethics committee of the Psychology Department at Ben-Gurion University of the Negev.

#### Apparatus and stimuli

Three pairs of possible and impossible objects were used as experimental stimuli (Figure 1). Possible and matched impossible objects were identical except for one (Figure 1A) or two features (Figures 1B,C) inserted to make the object’s global 3D structure impossible. All pairs were used in previous experiments (Freud et al., 2013b, 2015).

**FIGURE 2:**
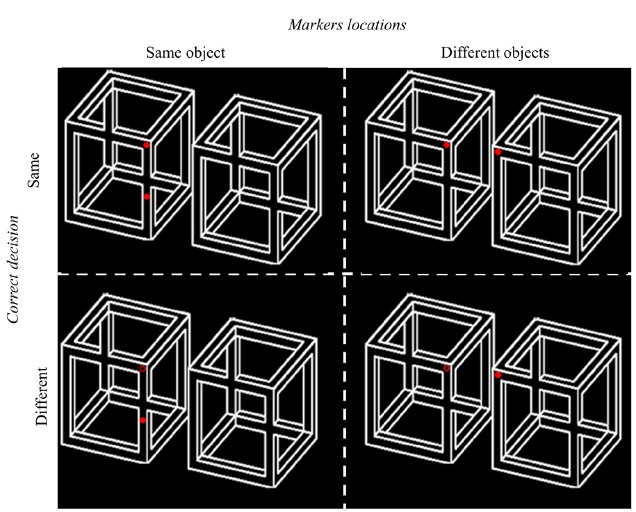
**Examples of stimuli presented in Experiment 1.** On each trial, two red circles were superimposed on the display and participants were asked to judge if the circles are same or different (rows). The locations of the circles varied across trials such that for half of the trials the circles were located on the same object (left column) and for the other half the circles were located on different objects (right column). Display orientation was counterbalanced across trials (see text for details).

In each trial, a pair of objects (possible or impossible) was presented (Figure 2). Stimuli subtended a visual angle of approximately 12° and were presented on a 19 inch computer screen (1024 × 768 resolution; refresh rate of 60 HZ). Additionally, two red circles-filled or empty (a visual angle-0.3° each) were superimposed on different locations on the objects depending on the specific experimental condition (same object/different objects). The circles were located on clear surfaces while avoiding more ambiguous locations such as object’s counters. Critically, the distance between the two circles was kept constant across trials regardless of their exact placement on the screen.

#### Design

Object possibility (possible/impossible), targets location (on different objects/on the same object), object orientation (horizontal/vertical; normal/mirror), and correct decision (same/different) served as within-subjects variables with each combination occurring on an equal number of trials (see Figure 2). The results were collapsed across object orientation and correct decision after preliminary analyses that ensured that these variables did not affect the interaction between possibility and target location [*F*s < 1]. Additionally, object exemplar served as a between-subject variable (three levels; Figure 1). Both accuracy and reaction times were recorded.

#### Experimental procedure

Participants were instructed to report as accurately and as quickly as possible whether the two red circles in each trial were identical (filled or empty) or different (one filled and one empty; Figure 2) regardless of their locations. Object type (possible/impossible) was not explicitly mentioned to avoid possible attentional confounds.

Following four practice trials in which feedback was provided that were discarded from further analysis, participants were presented with 256 experimental trials distributed equally and randomly between the different conditions. The trials were self-generated; each trial began with a fixation point (300 ms), followed by the experimental display (1000 ms) and a blank screen (1500 ms). Responses were recorded using a keyboard in which two stickers were attached to the designated response keys.

### RESULTS

Accuracy rate was high (93.6%). A repeated measures analysis of variance (ANOVA) revealed no accuracy-based effects of object type or of target locations [all *F*s < 1]. Analysis was therefore focused on RTs for correct trials.

**FIGURE 3:**
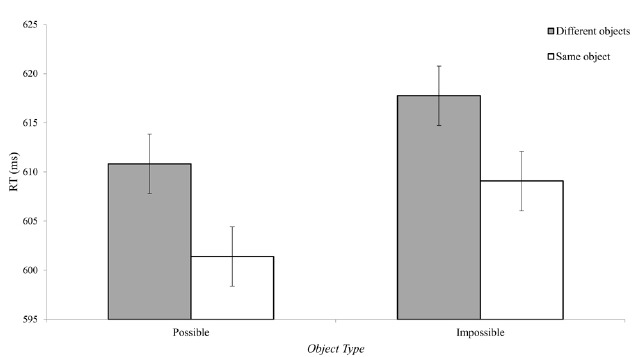
**Reaction times in Experiment 1.** Participants responded faster when the circles were located on the same object (an object-based attentional effect) regardless of object possibility. In addition, shorter reaction times were found for possible compared to impossible objects. Error bars represent confidence intervals as calculated for repeated measure ANOVAs for the effect of circles locations (Jarmasz and Hollands, 2009).

Latencies shorter than 200 ms and longer than 1000 ms were considered outliers and were removed from further analysis (4% of the correct responses). The reaction time data is presented in Figure 3. As can be seen, when targets were located on the same object, reaction times were faster compared to trials in which the targets were located on two different objects. A repeated measure ANOVA revealed a main effect of target location [*F*_(1,26)_ = 5.93, *p* < 0.05, ${\text{η}}_{\text{p}}^{\text{2}}$ = 0.18] with no interactions with object possibility [*F*_(2,26)_ < 1], or specific exemplar [*F*_(2,26)_ = 1.5, *p* > 0.2]. The three-way interaction was not significant [*F*_(2,26)_ = 1.6, *p* > 0.2]. Importantly, planned comparisons confirmed that the benefit of “same object” was evident for possible [*F*_(1,26)_ = 5.42, *p* < 0.05] and marginally for impossible objects [*F*_(1,26)_ = 3.85, *p* = 0.06].

Interestingly, despite the fact the impossible objects drew attention in a similar fashion to possible objects, a main effect was found for possibility, with slower reaction times for impossible compared to possible objects [*F*_(1,26)_ = 12.8, *p* < 0.001, ${\text{η}}_{\text{p}}^{\text{2}}$ = 0.33]. A marginal interaction was also found between exemplar and object possibility [*F*_(2,26)_ = 2.7, *p* = 0.08], indicating a more robust effect for some exemplars compared to others. Nevertheless, a similar trend of slower reaction times for impossible objects was found for all exemplars.

Finally, for each participants only one exemplar was used along the experiment to increase statistical power. A similar procedure was also used in previous studies that examined the sensitivity of the visual system to object impossibility (e.g., Shuwairi et al., 2007; Freud et al., 2014). However, repeated presentations of these objects might reduce their perceived impossibility. To examine this issue, we have reanalyzed that data separately for the first and the second part of the experiment. This analysis revealed that in addition to the main effect of object possibility [*F*_(1,26)_ = 14.6, *p* < 0.001], a main effect for experiment’s part was found [*F*_(1,26)_ = 29.6, *p* < 0.001], such that faster RT were found in the second part of the experiment compared to the first half. Importantly however, these two factors did not interact with one another [*F* < 1], suggesting that despite the repeated presentations of the same exemplar, the visual system did not treat the impossible object as different (i.e., more possible) at the second part of the experiment.

### DISCUSSION

The results of Experiment 1 show that impossible objects draw visual attention in a similar fashion to possible objects. This finding suggests that despite their unusual spatial structure, impossible objects share similar perceptual organization processes to those mediating the representation of possible objects. This same-object benefit is observed despite the relative complexity of the stimuli compared to previous studies (e.g., Moore et al., 1998; Zemel et al., 2002; Drummond and Shomstein, 2010). Moreover, for both object categories, the attentional capture occurred automatically with an object-based effect obtained despite the fact that the task was irrelevant to object structure (Kimchi et al., 2007).

One can argue that Gestalt cues such as common region, closure, etc., which are evident for both object categories, could account for the object-based effects observed for possible and impossible objects. We cannot fully disqualify this alternative. Still, a recent study indicates that a unified internal representation of an object, rather than a mere collection of its features, is required to support the object-based facilitation effect (Naber et al., 2011). Thus, the object-based effect found in Experiment 1 for impossible objects implies that object representations exists for impossible objects despite the spatial incoherency inherent to this object category.

Note that the two circles were located on the same 2D structurally possible surface. This was done to ensure that the perceptual requirements were equal between the possible and impossible conditions and that the circles were located on visually identical locations for the two object categories. Presentations of the circles on different surfaces for possible and impossible objects could have therefore resulted in an unwarranted confound which is avoided by the present design. However, this issue may raise an alternative explanation according to which the attentional selection was mediated by the processing of the 2D plane of the objects rather than of 3D information. Nevertheless, several lines of evidence are not in accord with such an alternative explanation. First, in the present study a main effect was found for object possibility, indicating that participants still processed object 3D information despite being irrelevant to the task in hand. Additionally, previous works from our lab and from other labs have shown that object 3D structure is processed in an automatic fashion (Goldfarb and Tzelgov, 2005; Freud et al., 2013a) and that object possibility is processed even when object category is irrelevant (Shuwairi et al., 2007; Regolin et al., 2011; Freud et al., 2014, 2015). Hence, it is unlikely that the participants independently processed the 2D plane in a unified manner, while other portions of the stimulus were not integrated.

In addition to the main result of similar object-based attention effects for the two object types, we also found some differences between the processing of the two object categories. In particular, slower classification times were found for impossible compared to possible objects. The nature of the underlying perceptual mechanisms that mediate the delayed responses for impossible objects was further investigated in Experiment 2.

## EXPERIMENT 2

The overall slower reaction times found for impossible objects in Experiment 1 could be accounted for by two fundamentally different plausible perceptual mechanisms. According to a bottom–up scheme, object structural impossibility can be encoded by a simple-to-complex architecture, based on hierarchical shape detectors. According to such an alternative view, the spatial ambiguity embedded in impossible objects impairs the initial perceptual organization of this object category. Only later in time along the hierarchy of visual processing, local valid depth cues are used to support object-based representation. Note, that previous research has shown that such models could account for the representation of complex stimuli such as faces and objects, and could also account for effect of global-configural organization (Jiang et al., 2006).

On the other hand, according to our working hypothesis, the initial representation of impossible objects is intact, and only later in time along the hierarchy of visual processing object impossibility is represented. Experiment 2 utilized a different experimental design aimed to distinguish between these two alternatives. To this end, we tested when along the processing hierarchy the perceived differences between the object categories start to emerge by manipulating exposure duration. We used two exposure durations. In the short exposure duration the stimulus was presented for 85 ms as previous studies suggested that this duration is the minimal time required for successful object recognition (Grill-Spector and Kanwisher, 2005; Sayres and Grill-Spector, 2006). The long exposure duration was set to 986 ms to allow full processing of object structure.

**FIGURE 4:**
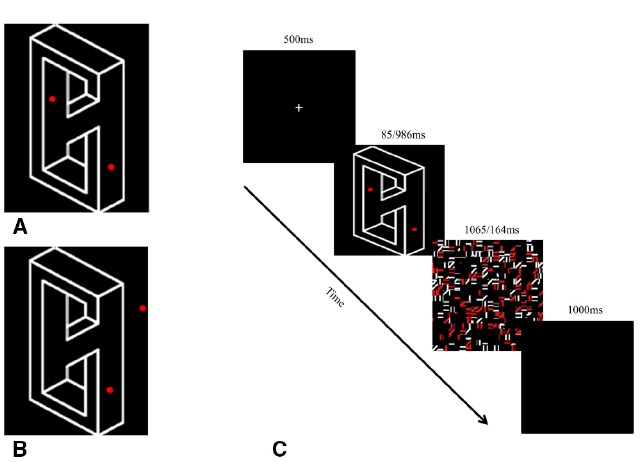
**Stimuli and experimental design Experiment 2.** On each trial, participants were asked to judge if the two circles are located inside the boundaries of the object **(A)**, or whether one of the circles is located outside its boundaries **(B)**. Exposure durations were manipulated to enable minimal processing of the object (85 ms) or full processing of the object (986 ms) **(C)**.

Importantly, in order to test object processing *per se*, we adopted a new task in which only a single object was presented in each trial. Participants were required to judge whether two circles are located on the object or whether one of the circles is located outside the boundaries of that object (Figures 4A,B).

According to our working hypothesis, the initial perceptual processes subserving the processing of possible and impossible objects should be similar and therefore no differences were expected to be found between the two object categories for short exposure durations. On the other hand, for the longer exposure duration, we predicted that there would be enough time for the incoherency of the structural information embedded in impossible objects to be processed, which should in turn impede the fine-grained representation of this object category.

### MATERIALS AND METHODS

#### Participants

Twenty six students (none of whom participated in Experiment 1) with normal or corrected to normal vision participated in the experiment. They all provided informed consent to participate in the experiment and received 5$ equivalent for their participation. The data of two participants was discarded from the analysis due to exceptionally low performance in the short exposure time condition (<50%).

#### Apparatus and stimuli

Seven pairs of possible and impossible objects were used in Experiment 2. Each trial display contained only one object that subtended a visual angle of approximately 9° visual angle, and was presented on a 19 inch monitor (resolution 1024 × 768; refresh rate of 60 HZ). Two red circles (visual angle: 0.3°) were superimposed at different locations on the objects depending on the specific experimental condition. For half of the trials, the circles were located inside the boundaries of the object, and for the other half, one circle was located inside the object and the other was located outside of its boundaries (Figure 4). Critically, the distance between the two circles was kept constant across trials regardless of their exact position on the screen.

#### Design

Object possibility (possible/impossible), exposure duration (85 ms/976 ms), object orientation (aligned /left/right mirror) and correct decision (in/out) served as within-subject variables with each combination occurring in an equal number of trials. The results were collapsed across object orientation and correct decision following a preliminary analyses which ensured that these variables did not interact with possibility or exposure duration [*F*s < 1]. Different exposure durations were presented in separate experimental blocks and the order of blocks was counterbalanced across participants.

An initial analysis of accuracy scores revealed a three-way interaction between exposure duration, possibility, and block order [*F*_(1,22)_ = 3.82, *p* = 0.06, ${\text{η}}_{\text{p}}^{\text{2}}$ = 0.14], implying a carry-over effect between blocks. To avoid this effect we discarded the second block of each participant and treated exposure duration as a between-subject variable. Importantly, the overall pattern of the results remains similar between the two analyses.

#### Experimental procedure

Participants were asked to judge whether the two circles are located on the object or whether one of the circles is located outside the boundaries of that object (in/out) (Figures 4A,B). The nature of the objects (possible/impossible) was not explicitly mentioned to avoid attentional confounds.

Following four practice trials in which feedback was provided, participants were presented with 448 experimental trials distributed equally and randomly between the different conditions. The trials were self-generated; each trial began with a fixation point (500 ms), followed by the experimental display (85/986 ms) and a mask that completed the display duration to 1150 ms (1065, 164 respectively). Trials ended with a blank screen (1000 ms; Figure 4C). Responses were given on a keyboard, similarly as in Experiment 1.

### RESULTS

The objective of Experiment 2 was to test whether the differences observed in Experiment 1 between possible and impossible objects were mediated by early or by late perceptual processes.

Accuracy and RT were analyzed using repeated measures ANOVA with possibility (possible/impossible) as a within-subject variable and exposure duration (85 ms/976 ms) as a between-subject variable. For the RT data, only a main effect of exposure time was observed. RTs were generally faster for the long exposure duration [*F*_(1,22)_ = 4.67, *p* < 0.05, ${\text{η}}_{\text{p}}^{\text{2}}$ = 0.17]. No effects were found for possibility or the interaction between exposure duration and possibility [both *F*s < 1].

**FIGURE 5:**
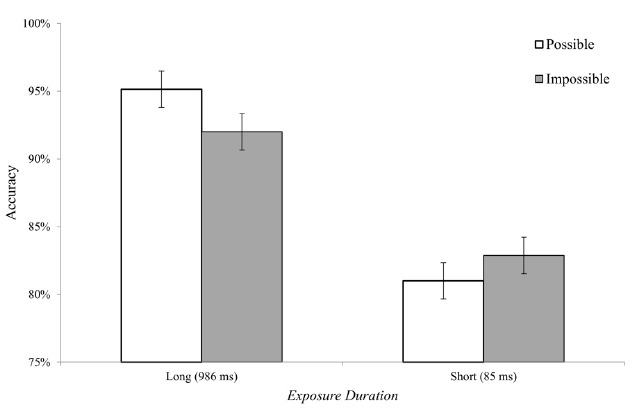
**Accuracy data for Experiment 2.** Differences between possible and impossible objects were found only for long (986 ms) exposure durations. For short exposure durations (85 ms) no differences were found between the two object types. Error bars represent confidence intervals as calculated for repeated measure ANOVAs for the effect of object possibility (Jarmasz and Hollands, 2009).

The ANOVA for the accuracy data revealed a significant main effect of exposure duration, with more accurate responses in the long exposure duration condition [*F*_(1,22)_ = 13.74, *p* < 0.01, ${\text{η}}_{\text{p}}^{\text{2}}$ = 0.38]. More importantly, a significant interaction was found between possibility and exposure durations [*F*_(1,22)_ = 5.43, *p* < 0.05, ${\text{η}}_{\text{p}}^{\text{2}}$ = 0.2]. Planned comparisons reveled that for the short exposure duration, there were no differences between possible and impossible objects [*F*_(1,22)_ = 1.88, *p* > 0.15]. On the other hand, for the long exposure duration, performance was more accurate for possible compared to impossible objects, as indicated by the marginally significant planned comparison [*F*_(1,22)_ = 3.69, *p* = 0.06; Figure 5]. Importantly, the accuracy rate in the short exposure duration was above 80% and therefore the lack of difference between object categories could not be accounted by a general lack of sensitivity (i.e., floor effect).

Finally, to ensure that in the short exposure duration participants did not apply a strategy of attending one circle while guessing about the location of the second circle, we computed a sensitivity measure (*d*′ score), that took into account response bias separately for possible and impossible objects. Correct responses for the “in” condition (i.e., two circles that were located on the object) were defined as hits, while incorrect responses for the “out” condition (i.e., one circle which was located outside the object) were defined as false alarms. These scores were subjected to a repeated measure ANOVA. A significant interaction was found between possibility and exposure duration [*F*_(1,22)_ = 4.24, *p* = 0.05, ${\text{η}}_{\text{p}}^{\text{2}}$ = 0.16] and planned comparisons showed that the sensitivity was significantly higher for possible objects [*d*′_(possible)_ = 3.49, *d*′_(impossible)_ = 3.19] in the long exposure duration [*F*_(1,22)_ = 4.58, *p* < 0.05], while no significant differences were observed for the short exposure [*F*_(1,22)_ < 1; *d*′_(possible)_ = 1.96, *d*′_(impossible)_ = 2.06]. Importantly, the criterion, which reflects a response bias, was not different from 0 regardless of exposure durations [*F*s < 1].

These findings indicate that the perceptual differences between possible and impossible object arise at late perceptual stages while that at initial perceptual stages, the processing of possible and impossible objects is performed in a similar fashion.

## GENERAL DISCUSSION

The results of the current study showed that the representations of possible and impossible objects rely on similar initial perceptual processes, while differentiation between object categories emerge only later along the processing hierarchy.

Experiment 1 demonstrated that impossible objects draw attention automatically and similarly to possible objects, suggesting resemblance in the perceptual processes that mediate the representation of the two object types. Yet, differences in performance were also found between possible and impossible objects, with overall longer reaction times for impossible objects. Previous studies from our lab already showed that possible and impossible objects share similar a initial processing scheme (Freud et al., 2013a,b). In Experiment 1 we validated and extended these findings using a different, well-established paradigm of object-based attention. Importantly, while previous studies required overt judgments of object dimensions (e.g., size of a particular dimension), here the objects were task-irrelevant and therefore enabled testing whether attention can be allocated in an automatic fashion even for impossible objects.

Experiment 2 was aimed to unravel the source of the overall longer reaction times that were found for impossible objects in Experiment 1. The results indicate that the differences between possible and impossible object emerge only when objects were presented for long exposure duration, and therefore are probably affected by late perceptual processes, such as the rendering of fine-detailed description of object structure (Hochstein and Ahissar, 2002). Importantly, the level of processing is inevitable, and participants could not “choose” to process only the gist of the object. Presumably, in the long exposure duration the visual system automatically attempted to generate a fine-detailed description of the impossible objects, however the incoherency of 3D structure could have impaired this process, and therefore the task (which relied on the processing of the object) was performed less accurately compared to situations in which the fine-grained representation could be successfully generated. These results converge with a recent study from our lab that used the Garner’s speeded classification task to unravel the perceptual representation of possible and impossible objects. In this study the level of processing was manipulated by filtering-out specific spatial frequency ranges and not by limiting exposure duration as in Experiment 2. Interestingly, it was found that when low spatial frequency (LSF) information, which usually supports rapid, coarse representation (e.g., Bar et al., 2006), was available, similar processing style was observed for possible and impossible objects. On the other hand, when LSF information was filtered out, only HSF information was available which could support late, but detailed processing of object shape. Under this condition, a dissociation was found between the object categories. Specifically, possible objects were still processed in a holistic unified manner, while impossible objects were no longer processed as unified objects (Freud et al., 2014).

The investigation of the representation of impossible objects could shed light on the cognitive and neural mechanisms that mediate the perception of novel (possible) objects in everyday life. In particular, the results of the present study could be taken as a general support for the reverse hierarchy theory (RHT; Hochstein and Ahissar, 2002) in the context of 3D information representation. In a nutshell, the RHT theory proposes that visual perception is based on two main stages. First, a feed-forward processing stream represents the “gist of the scene” while the exact details are not fully encoded. The second stage is based on recurrent processing that relies on low-level regions in the visual cortex and enables the encoding of detailed information.

According to the RHT, object-based attention is accomplished by fast feed forward processing. Thus, the similar gist, which characterized matched possible and impossible objects, could have mediated the equivalent object-based attention results obtained in Experiment 1. Similarly, in Experiment 2, when objects were presented for a short duration, the task could have been performed based on the gist of the objects while the fine details were not accessible. This could account for the similar performance patterns obtained for the two object categories under this condition. On the other hand, when objects were presented for longer durations, feedback processing could have taken place and the fine details that define spatial ambiguity of impossible objects could be processed, which in turn led to reduced accuracy for impossible objects.

The perceptual processes that mediate the representation of impossible objects could also be discussed in light of the Bayesian framework. According to this framework, information conveyed by the stimulus is represented by the visual system as a conditional probability density function. The visual system uses past experience to compute the most probable interpretation given the retinal image stimulus (Kersten et al., 2004; Knill and Pouget, 2004). This conceptualization was developed by behavioral and computational studies that demonstrated that Bayesian models nicely fit to the perceived experience in a variety of perceptual phenomena, including depth perception which follows the integration of multiple depth cues (e.g., van Ee et al., 2003) and visual illusions of motion (Geisler and Kersten, 2002; Weiss et al., 2002). Although the present investigation was not aimed to test whether a Bayesian framework could account for the representation of impossible objects, it is plausible that the visual system erroneously estimates the likelihood of different depth cues (global/local) which could lead to an incoherent percept of the stimulus. Such a process may also mediate the behavioral advantage observed for possible objects in Experiment 1 and in the long exposure duration of Experiment 2. Future studies could use this framework to further unravel how the brain estimates and weights different types of monocular depth cues in a Bayesian fashion.

To summarize, our findings suggest that initially, possible and impossible objects are spatially organized in a similar manner and that impossible figures are represented in a similar manner to possible ones at least in terms of capturing automatic visual attention. The visual system successfully utilizes intact shape attributes to create a successful representation of impossible objects. These properties override the odd spatial layout inherent to impossible objects while the phenomenological differences between object-types may reflect high-level perceptual processes that occur later in time. Yet, the nature of these processes needs to be elaborated by future research that will examine their dynamics and the cognitive and the neural correlates mediating these processes.

### Conflict of Interest Statement

The authors declare that the research was conducted in the absence of any commercial or financial relationships that could be construed as a potential conflict of interest.
